# Empowering citizens in international governance of nanotechnologies

**DOI:** 10.1007/s11051-015-3019-0

**Published:** 2015-05-12

**Authors:** Ineke Malsch, Vrishali Subramanian, Elena Semenzin, Danail Hristozov, Antonio Marcomini, Martin Mullins, Karena Hester, Eamonn McAlea, Finbarr Murphy, Syed A. M. Tofail

**Affiliations:** Malsch TechnoValuation, Vondellaan 90, 3521 GH Utrecht, The Netherlands; Department of Environmental Sciences, Informatics and Statistics, Ca’Foscari University of Venice, Dorsoduro 3246, 30123 Venice, Italy; Department of Accounting and Finance, Kemmy Business School, University of Limerick, Limerick, Ireland; Department of Physics and Energy, and Materials and Surface Sciences Institute (MSSI), University of Limerick, Limerick, Ireland

**Keywords:** Nanotechnology, International governance, Responsible research and innovation, Sustainability

## Abstract

The international dialogue on responsible governance of nanotechnologies engages a wide range of actors with conflicting as well as common interests. It is also characterised by a lack of evidence-based data on uncertain risks of in particular engineered nanomaterials. The present paper aims at deepening understanding of the collective decision making context at international level using the grounded theory approach as proposed by Glaser and Strauss in “The Discovery of Grounded Theory” (1967). This starts by discussing relevant concepts from different fields including sociological and political studies of international relations as well as political philosophy and ethics. This analysis of current trends in international law making is taken as starting point for exploring the role that a software decision support tool could play in multi-stakeholder global governance of nanotechnologies. These theoretical ideas are then compared with the current design of the SUN Decision Support System (SUNDS) under development in the European project on Sustainable Nanotechnologies (SUN, www.sun-fp7.eu). Through constant comparison, the ideas are also compared with requirements of different stakeholders as expressed during a user workshop. This allows for highlighting discussion points for further consideration.

## Introduction

Observation and participation in dialogue on the international governance of nanotechnology and other emerging technologies since the 1990s have inspired a search for concepts to describe the emerging governance structures as well as for setting ethically sound targets for consolidation of these governance structures. Examples of proposed concepts include nanoethics, responsible nanoresearch, nanosafety, precaution, stakeholder dialogue and governance of uncertain risks of nanomaterials. A major criticism of these proposals and initiatives is their arbitrariness: What distinguishes nanotechnology so categorically from other emerging technologies to call for nano-specific ethical principles or governance structures? A decade of research in nanoethics and ethical, legal and societal aspects (ELSA) of nanotechnology and of public and stakeholder dialogue has not resulted in a satisfying answer to this question.

The present article therefore starts from the other end, taking a particular theory of ethically sound global governance as a starting point and then considering its applicability to the case of international governance of emerging technologies including nanotechnology in a secondary instance. The core of the tentative theoretical framework is constituted by Risse’s (2002) review of the role of transnational actors in international governance, Rawls’ (1999) ideas on deliberative democracy and Habermas’ (2011) discussion of Kant’s ideal institutionalisation of “perpetual peace” (Kant 1795). Central research questions are as follows:

- What can theories of international relations and deliberative democracy contribute to understanding multistakeholder governance of emerging technologies?
- What role could software decision support play in democratising such governance?

## Grounded theory based analytical approach

The present article aims to contribute to the formulation of a substantive grounded theory [as proposed by Glaser and Strauss (1967)] for the field of international governance of nanotechnology through identification of conceptual categories and their conceptual properties and the formulation of generalised relations among the categories and their properties in an integrated form. Grounded theory is a good “fit” for this field, because we are dealing with an relatively new set of inquiries. Whilst the area of applied ethics has a substantial body of theory this sub-field does not. Hence, the need to return to primary sources to build theory up as it were.

According to Glaser and Strauss, the concepts should be analytic—revealing characteristics of the studied entities—and sensitising—yielding meaningful pictures. The formulation of such grounded theory starts with observation and participation in the field of study, in this case the emerging heterogeneous community of stakeholders interested in international governance of nanotechnology. This paper’s authors have been engaged in several research projects on the scientific, ethical, legal and societal (ELSA) and environment, health and safety dimensions of nanotechnology and this experience informs the interpretive lens applied to weave together interdisciplinary theoretical concepts from sociology, ethics and political philosophy with quantitative data on the international governance of nanotechnology from a variety of sources including interviews, workshops, field notes, literature research and surveys. The present article introduces concepts from established theories in international relations and philosophy to analyse data collected from different sources on current practices in international governance of nanotechnology. Glaser and Strauss (1967) promote such an introduction of theoretical concepts after the first round of data collection in generating a new grounded theory, provided that these concepts are relevant and that they fit the analysed data. The present article diverges from the conventional generation of a grounded theory in the sense that the latter is a theory in social sciences while the present article combines normative philosophical with descriptive sociological concepts in an interdisciplinary approach.

Glaser and Strauss (1967) explain that constant comparison combines explicit coding of data with constant redesign and reintegration of theoretical notions while reviewing the data. The aim is to generate a theory that is integrated, consistent, plausible, close to the data and readily operationalised for testing in quantitative research. It is not useful for provisional testing, because the data are not extensive enough nor coded extensively enough. The constant comparison method consists of four stages:

1. Comparing incidents applicable to each category;
2. Integrating categories and their properties;
3. Determining the theory, that should be parsimonious in variables and formulation and as wide as possible in scope; and
4. Writing the theory

The analyst can go back and forth between the stages as more data are collected and analysed.

Our data sources comprise (a) literature on global governance of nanotechnology (e.g. scholarly articles published in the journal Nanoethics since 2007,^1^ policy and stakeholder discussions reviewed in Malsch 2011; Malsch et al. 2012), (b) Interview with stakeholders from the regulatory, industrial and insurance sectors within the context of nanosafety projects like EU FP7 SUN and SANOWORK, and (c) transcript of a workshop conducted in the frame of SUN project on the potential role of decision support in the international governance of nanotechnology. These data sources are replete with evidence that scholars and policy makers interested in responsible nanotechnology development have introduced and tested numerous concepts and tools for putting such global governance of nanotechnology and other emerging technologies into practice. Along with providing sources to triangulate during grounded theory development (Patton 1999), these data sources highlight the relevance of international nanotechnology governance theories.

Our analysis is structured as follows. The section “Theoretical concepts on global governance of nanotechnology engaging multiple stakeholders” considers some theoretical concepts relevant to the global governance of nanotechnology, and how these concepts relate to the current global governance of nanotechnology. “Role of Decision Support in Global Multi-Stakeholder Governance of Nanotechnology during Norm Creation” section outlines the potential role of decision support in the global governance of nanotechnology. “Grounded theory for international governance of nanomaterials” section integrates these discussions to the notion of international governance of nanotechnology based on the grounded theory.

## Theoretical concepts on global governance of nanotechnology engaging multiple stakeholders

The issue of the participation of different state and non-state actors in global governance has been the topic of lengthy scholarly debates in different fields including sociological and political studies of international relations as well as political philosophy and ethics. Some relevant concepts and models are discussed below.

### Sociological and political scientific aspects of global governance of nanotechnology

*Who are the actors* involved in international governance in addition to states? Risse’s (2002) review of transnational actors and world politics analyses the state of knowledge on the question when and under which conditions trans-national Actors (TNA) or networks matter in international governance in addition to states and international organisations. He distinguishes (non-governmental) advocacy networks diffusing norms (International Non-Governmental Organisations) and epistemic communities (Haas 1992) diffusing causal knowledge, as well as multinational companies (c.f. Hanekamp and Wütscher 2007).

Haas (1992) defined ‘epistemic communities’ as…” network[s] of professionals with recognised expertise and competence in a particular domain and an authoritative claim to policy-relevant knowledge within that domain or issue area. Although epistemic communities may consist of professionals from a variety of disciplines and backgrounds, they have the following:

1. A *shared* set of normative and *principled beliefs*, which provides a value-based rationale for the social action of community members
2. *Shared causal beliefs*, which are derived from their analysis of practices leading or contributing to a central set of problems in their domain and which then serve as the basis for elucidating the multiple linkages between possible policy actions and desired outcomes
3. *Shared notions of validity*—that is intersubjective, internally defined criteria for weighing and validating knowledge in the domain of their expertise
4. *A common policy enterprise*—that is a set of common practices associated with a set of problems to which their professional competence is directed presumably out of the conviction that human welfare will be enhanced as a consequence”.

The major dynamics of epistemic policy coordination are uncertainty, interpretation and institutionalisation (Haas 1992, p. 3). Concepts like epistemic communities and epistemic cultures (Knorr-Cetina 1999) have been widely used in social studies of science. Davis Cross (2013) criticises the restrictive application of the concept in subsequent literature, limited to single case studies and groups consisting only of scientists. She advocates a more flexible interpretation of the concept, improving its utility for understanding how knowledge translates into power in international relations.

Especially, the concept of epistemic communities as defined by Haas reveals relevant dynamics in the case of international governance of emerging technologies as these technologies introduce uncertain risks to the agenda of international policy making the interpretation of which calls for scientific expertise and that may lead to new institutionalisation of international governance regimes (c.f. IRGC 2006). Klaessig (2014) applies this concept to critically analyse international discussions developing official practices for nanoEHS (Environment, Health and Safety aspects). This is characterised by uncertainty not only about risks of nanomaterials and widespread differences in interpretations of definitions but also about suitable methodologies and interpretation of results of scientific experiments among scientists and policy makers. Klaessig raises issues about the current process of institutionalisation of what he calls “official science” replacing normal science, because this mixes scientific quality standards with political considerations and excludes some results of scientific experiments on arbitrary grounds.

**Fig. 1:**
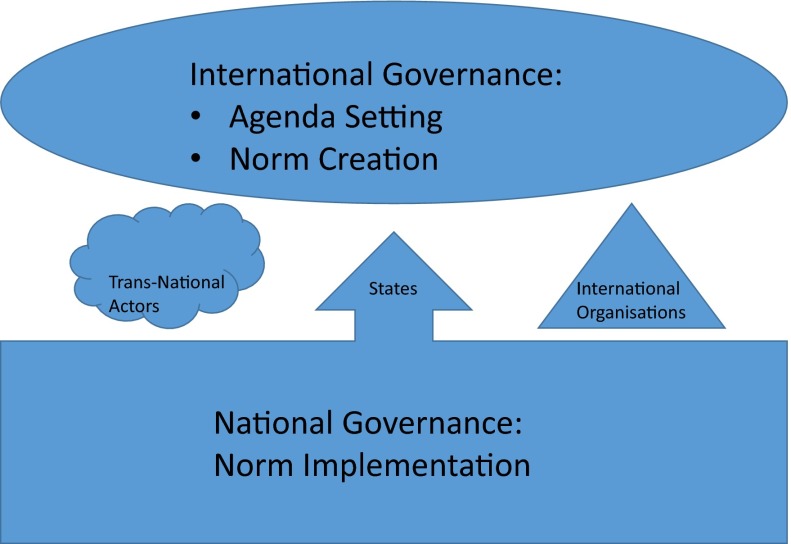
The actors involved in international governance following Risse (2002)

*What role do these actors play?* Studies have demonstrated that transnational actors including epistemic communities and advocacy networks can have a substantial impact on state policies, on the creation of international norms and on the diffusion of these norms into domestic practices. It is unclear why and under which conditions, due to the lack of case studies of failed campaigns, according to Risse (2002). Risse et al. (1999) argues that international institutions provide arena’s in which the activities of transnational actors are allowed to flourish, including the EU, World Bank and UN World Conferences. Following Risse (2002) three phases can be distinguished in the international policy cycle (Fig. 1):

- Agenda setting, where the influence of transnational actors has always been the greatest. In the case of the risk governance of nanomaterials, this is demonstrated by the Canadian-based international NGO ETC group (Erosion, Technologies and Control). This is certainly responsible for setting the agenda for the debate on risk governance. Already in 2003 it pointed to the potential environmental and safety risks of “Green Goo” and converging technologies and called for a moratorium and a precautionary approach (ETC Group 2003a, b, c, d, e, f, 2004). Their call was picked up by UK Prince Charles and motivated the Royal Society and Royal Academy of Engineering study on nanotechnology in the UK (Dowling 2004) as well as a wide range of studies on ethical and societal aspects of nanotechnologies, EHS aspects of nanomaterials and public and stakeholder dialogue initiatives worldwide (reviewed in Malsch 2011).
- International norm creation, dominated by national governments and international organisations. Risk governance of nanomaterials is currently in this stage of international norm creation. Roughly in the period 2003–2012, nanotechnology was probably the first research area where broad and public stakeholder dialogue on such international norm creation was experimented with, engaging epistemic communities, multinational companies and their associations as well as international NGOs and trade unions. Currently, in addition to national governments and international organisations, such engagement of transnational actors is mainly restricted to epistemic communities and multinational companies, typically behind closed doors, in inter-, trans- and national standardisation bodies and other expert committees (reviewed in Malsch et al. 2012).
- Norm implementation, in which evidence suggests that transnational actors and epistemic communities assume centre stage. The legalisation process legitimises their positions and they assume a monitoring role International Organisations who have to remain neutral cannot perform. It is too early to say what role transnational actors will eventually play in the implementation of the nanosafety norms. This is because the legislative framework for nanomaterials is still under development, and may theoretically still result in a new “Lex Specialis” for handling the chemical identity of these materials on the market, in the words of a policy maker. However, the main trend in the international norm creation dialogue on nanomaterials appears to aim at incorporating nanomaterials in current legislation in four phases: occupational health and safety, chemicals, consumer products and environment. The consumer products are covered by product specific legislation for cosmetics, food, pharmaceuticals and medical devices, biocides etc. (Malsch et al. 2015). Transnational actors could therefore continue to play the same roles in implementation of the existing legislation in each phase, provided they are made aware of the specific properties of nanomaterials.

### Ethics and political philosophy aspects of global governance of nanotechnology

According to Risse (2002), the main unresolved issues in the literature on transnational actors are “*How can global governance* by increasingly complex tripartite networks *solve the dual problem of ensuring ‘input legitimacy’* of those concerned by the legislation *and ‘output legitimacy’* through effective and enhanced problem solving? Closer examination of the discussions on responsible governance of nanotechnology (e.g. Malsch 2011) reveals a third unresolved issue: *“how to govern the emergingtechnology responsibly during the period of international norm creation?”* Despite the fact that this is only a temporary issue, it is not trivial: The issue of uncertain risks of nanomaterials has entered the international agenda in 2003, but insiders expect final agreement on common norms to take another 5–10 years. During the 20 years in between, more and more products incorporating nanomaterials are entering the market. And this is only one example of the continuous stream of emerging technologies. The following sub-sections advance theoretical arguments on the issue of input legitimacy and output legitimacy of global governance of technologies, moving on to consider the issue of global governance during norm creation. The role of the insurance sector in the international norm creation phase is considered as an example of the role played by this stakeholder in international norm creation.

#### Input legitimacy: democratising decision making to all stakeholders

The issue of input legitimacy is the focus of the so-called ‘democratic deficit debate’. Rawls (1971, 1999), Habermas (1996, 1998) and others have proposed deliberative democracy as a solution.^2^

Rawls’ approach to deliberative democracy is a non-linear, co-constructive approach to decision making. It is based on deliberative democracy and the participation model of pure procedural justice.^3^ Rawls does not agree with acting on the basis of calculated probabilities (risk assessment). He says we have to add on the condition that substantial *values* are at stake in the choices we make when there is the risk of suffering harm or loss depending on the decision taken. Rawls’ approach to deliberative democracy is founded on four key commitments.^4^

Rawls’ theory of Justice on which his approach to deliberative democracy is grounded is founded on “justice as fairness”.^5^ “It defines the conditions under which the spontaneous coherence of the aims and wants of individuals is neither coerced nor contrived but expresses a proper harmony consistent with the ideal good” (Rawls 1971). The principles which emerge from the original position apply to the basic structure of society hence the approach can be developed as a *legitimate input* to strengthen collective decision making about the assignment of rights and duties; to develop norms in the context of the distribution of social and economic advantages in different types of situations and to contribute to multi-stakeholder global governance.

The “original position” is individualistic from which evolved “Political Liberalism” (Rawls 1996) which accepts the “fact of reasonable pluralism”, the fact that a diversity of reasonable yet conflicting and irreconcilable religious, philosophical or moral doctrines can be affirmed by citizens in the free exercise of their capacity for a conception of the good. Persons are assumed to have two moral powers—a capacity for a sense of justice and for a conception of the good. Rawls argues that citizens in a constitutional democracy who hold opposing even irreconcilable conceptions of the good can find a shared basis of reasonable agreement through an “overlapping consensus” on the basis that citizens possess virtues of tolerance, readiness to meet others half way, reasonableness and principles of fairness. Their comprehensive doctrines are generally not fully comprehensive in so far as they will develop an allegiance to the concepts that help to bring about consensus.

The aim is to develop a criterion of justice that would be agreed upon by all under conditions that are fair to all; which could be used to assess the fairness of the institutions of society, to structure moral discussions while recognising the plurality of incompatible and irreconcilable moral frameworks in a democratic society and to justify the outcome of those discussions.

**Fig. 2:**
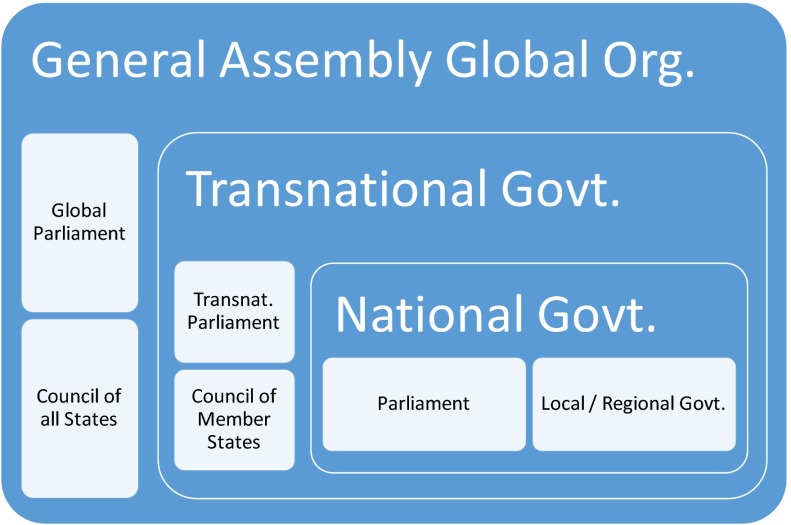
Combining global and national citizenship following Habermas (2011)

Habermas (2011) takes a more utopian ideal as a starting point for his philosophical analysis than the practical issue of ensuring input legitimacy. In an essay on Kant’s idea of perpetual peace, Habermas (2011, pp. 21–62) explains that Kant (1795) introduced the concept of the law governing global citizenship in addition to constitutional and international public law. According to Kant, the persons obeying the law must also be co-legislators, not only within a particular state, but also at global level. Kant expects that this will result in peace. Habermas sees conceptual problems in Kant’s ideal, and notes that it is incompatible with our historical experiences, but this discussion goes beyond the scope of the present article. It suffices to note that his analysis of Kant’s ideas inspires Habermas (2011, pp. 176–195)^6^ to sketch a global three layered system of national, transnational and international government with two legitimisation channels (Fig. 2):

1. From global citizens through the international community to peace and human rights politics of the global organisation
2. From state citizens via their national states and possibly the appropriate regional regime to the *transnational negotiation system, that carries responsibility in the framework of the international community for world internal political issues*
3. Both channels meet in the General Assembly of the global organisation, that is responsible for the interpretation and further development of the political constitution of the global society and therefore for the normative parameters of both peace and human rights politics as well as world-internal politics (Habermas 2011).

At global level, the central forum for the current international norm creation on nanomaterials, an example of a world internal political issue, is arguably the Organisation for Economic Cooperation and Development OECD where the working party on manufactured nanomaterials is preparing decision making by the Council of Ambassadors of OECD Member States.^7^ “Recent decisions relevant to nanomaterials are the OECD Council Recommendation on Nanomaterials of September 2013, which is not legally binding. The OECD Council Decision on MAD (Mutual Acceptance of Data) is legally binding for chemicals in general. The aim of the regulatory discussions at OECD level is to clear the way for nanomaterials to become part of this international system of legally binding agreements on the exchange of safety data” (Malsch et al. 2015). Several UN bodies coordinate their discussions on nanomaterials through the Inter-Organisation Programme for the Sound Management of Chemicals (IOMC).^8^ Nanotechnologies and manufactured nanomaterials are among the IOMC and the strategic approach to international chemicals management (SAICM)^9^ Emerging Policy Issues. The IOMC participating organisations OECD and the United Nations Institute for Training and Research (UNITAR) are taking care of this issue. UNITAR focuses on training and capacity building of governments in developing countries and has published a pilot “Guidance for Developing a National Nanotechnology Policy and Programme” in 2011.^10^

This three layered system constitutes an institutionalised solution to the input-legitimacy problem formulated by Risse as it grants citizens a formal role participating in democratic decision making at all three levels. From Habermas’ perspective the deliberations in the IOMC, SAICM, OECD, UNITAR, and other participating international organisations and representatives of states would fit in the second legitimisation channel. What is missing is transparency of these deliberations to citizens of the participating countries. The endpoint of this international norm creation process should ideally be democratic co-decision making at the level of the UN General Assembly and an as yet non-existing global parliament of elected representatives of all world citizens. At transnational level in the European Union, the European Parliament and the Council of Ministers of Member States are already co-responsible for adopting or revising most European regulations and directives. Therefore the democratic deficit is less pressing at this level, hence the focus of this article is at the global level.

What remains to be clarified is what role transnational actors including advocacy networks, epistemic communities and multinational companies should play in this proposed system. Are they undesirable aberrations of the current imperfect structure of international relations, or can they be transformed into efficient and democratic representatives of global citizens? In addition, the issue of output-legitimacy remains unresolved in Habermas’ system.

#### Output legitimacy: advancing shared global goals?

Indicators for output-legitimacy of such an international governance system are effective and enhanced problem solving. Kant (1795) formulated the ideal of a global republic where global citizens constitute global law that imposes restrictions to the internal and external sovereignty of states. Kant introduced a pragmatic intermediary step in the form of a non-binding voluntary league of nations of republics that gradually expands itself. Habermas asserts that the UN and EU already are communities of states and citizens: the UN through its responsibility to protect international security of states and human rights of citizens, and the EU by its constitution. The problem is not the lack of a constitution, but the lack of means of enforcement (Habermas 2011). In this enforcement endeavour, transnational actors may again be granted a formal role, like the one they currently informally play in monitoring national implementation of international norms (Risse 2002). For nanomaterials, it is currently too early to see what role the interested transnational actors may fulfil after agreement on such norms will have been reached.

#### Governance of nanotechnology during international norm creation

Currently more pressing for nanomaterials is the third unresolved issue: “how to govern the emerging technology responsibly during the period of international norm creation?” Emerging technologies introduce uncertainty into the global governance regime because they may or may not be covered by the existing body of positive law. International governance mechanisms are adapted to reducing this uncertainty, formulating and adopting adequate legal instruments. The issue of short- to medium term solutions for governing uncertain risks of emerging technologies is at the core of the contemporary international dialogue on governance of nanomaterials. Several solutions have been proposed and implemented over the last decade, but consensus on a common solution is still lacking (e.g. Malsch 2013).

If the law turns out not to be the right medium for solving this short term issue in international governance, would ethics or morals be able to play a subsidiary role? Scherer et al. (2007) discuss what they call the “republican business ethics model” advocating a supplementary function of ethics within the framework of positive law. Business ethics is complementary to positive law where its rules fail to resolve emerging problems of business. They refer among others to Habermas’ (1996, 1998) political philosophy: The citizen and the corporation have double roles as private citizens and as citizens of the state or a community. The ISO international standard 26,000 for social responsibility of business and organisations (ISO 2010) is an example of such complementary business ethics.

Differences in political philosophies between the participants in the international dialogue on emerging technologies also influence their ability to consider ethical in addition to legal norms as governance instruments. A liberal perspective (e.g. Rawls 1971, 1999) tends to emphasise legal rights for citizens who have handed over responsibility for law making and law enforcement to the sovereign state they are in a social contractual relationship with. A communitarian perspective (e.g. MacIntyre 1981; Michael 1982; Walzer 1983; Taylor 1985) tends to emphasise moral rights and obligations engaging all citizens in a common responsibility for human rights, society and the environment.

Habermas argues that communitarianism is based on the assumption that individual or subjective rights are innate. “The alternative between ‘individualists’ and ‘collectivists’ disappears when one adopts the unity of individuation and specialisation processes as fundamental legal principles. Because legal persons can also merely develop themselves as individuals through the process of socialisation, the integrity of the individual person can only be protected by simultaneously guaranteeing free access to the interpersonal relations and cultural traditions within which they can maintain their identity. A well-understood individualism remains incomplete without this addition of communitarianism” (Habermas 2011, p. 76).

Malsch (2011, 2013, forthcoming) discusses how a communitarian perspective may shed new light on the dynamics in and dialogue about ethical and societal aspects of nanotechnology. This perspective allows for observing as well as legitimising the roles played by non-state actors in national and international governance of emerging technologies. Communitarianism does not offer tools for differentiating different types of non-state actors. Risse’s discussion of the roles of transnational actors distinguishing advocacy networks or International Non-Governmental Organisations (INGO), epistemic communities and Multinational Companies (MNC) does facilitate such tools for describing the dynamics of international governance of emerging technologies. Habermas’ discussion of the ideal organisation of global and national citizenship offers tools for investigating the normative, prescriptive side of the issue.

As an intermezzo in these top-down theoretical considerations the next section takes a bottom-up perspective on governance during international norm creation by zooming in on the role played by the insurance sector as an intermediary between regulators, industry and other stakeholders.

### Nanotechnology sustainability from the perspective of insurability

Concerns surrounding the health risks of engineered nanomaterials from public interest groups, the lack of suitable regulation, an environment of legal uncertainty driven by ever changing legal definitions of injury or even “nanomaterial”, and the lack of specifically tailored insurance products targeted at the occupational or consumer risks from nanotechnologies are putting the industry’s long-term economic viability at risk. There is interdependence between risk perceptions, regulation, the legal profession and insurability; in this emerging and fast changing space, regulators are failing to keep pace and hence risk perception among the above stakeholder groups remains a stubborn problem. In the absence of well-developed regulatory protocols, the insurance industry has for example come to occupy a key role as an effective lobby in terms of improved occupational risk management practice. The failure of regulators and prominent industry participants to create uniform standards for nanomaterials creates an environment of uncertainty for all stakeholders. The latter not only causes problems in term of risk transfer but may lead to ill-conceived regulatory requirements, potentially exhausting resources and stifling innovation in the sector. In the absence of targeted regulation and standards, the insurance industry will continue to do what it has always done: It will insure uncertain risks based on risk appetite and a careful consideration of worst case scenarios, although in the short term this will likely entail high insurance premiums, reflecting the cost of uncertainty. In this regard insurers effectively act as proxy regulators and beacons of assurance to those individuals and groups who share concerns about the human and environmental threats they perceive coming from emerging technologies.

**Fig. 3:**
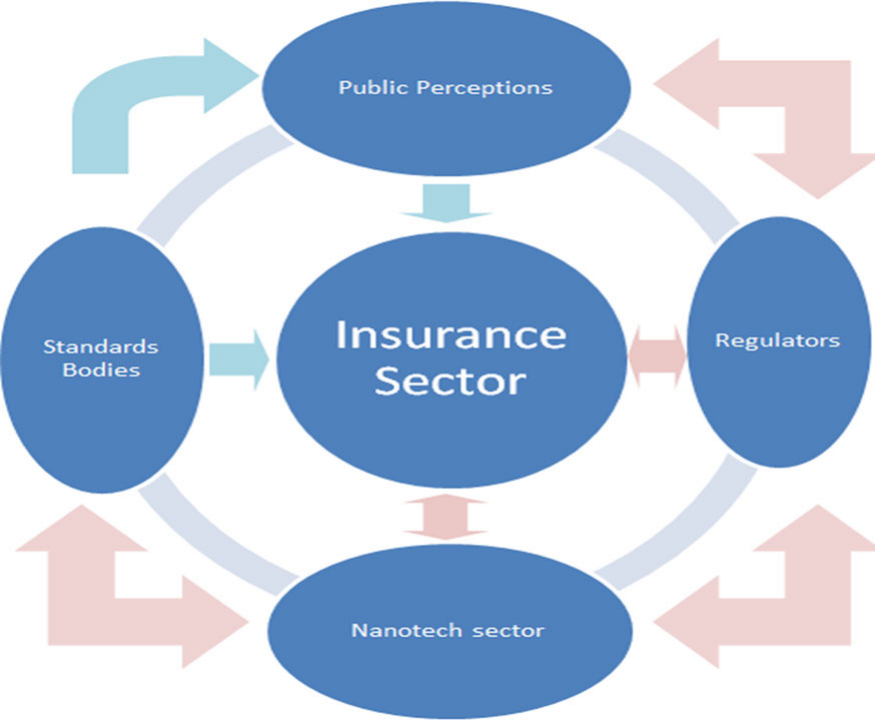
Relationships among key centres of civic influence and decision-making that mutually effect and benefit one another

Figure 3 illustrates how key centres of civic influence and decision-making can mutually effect and benefit one another through multiple self-reinforcing feedback loops. For example, this can happen through the sharing of risk information and proposals for standard operating procedures (SOPs) etc.

It can be argued that insurers play a crucial role in defining and shaping these relationships. For example, the provision of insurance acts as a proxy regulator for official regulators by signalling that the insurability of risks renders them manageable and therefore amenable to regulation. This would tend to have positive impacts on public perception that would only be of benefit to insurers. For example, insurers involved in litigation are sometimes at the mercy of juries whose members’ opinions reflect the prevailing views of the wider public in relation to perceived risks. Generally speaking, the effects that insurers set in motion are such that they eventually reinforce and support the positions they have taken, although there are exceptions, for instance, insurers’ long history with asbestos litigation.

## Role of decision support in global multi-stakeholder governance of nanotechnology during norm creation

As the theoretical analysis indicates, international governance of emerging technologies is a process in which a variety of transnational actors participate in cooperation with states and international organisations. This realm is increasingly being institutionalised and norms are being created and revised to accommodate technological progress and other contextual changes. Though proposals for improving input and output legitimacy as well as governance during the norm creation phase have been made and are being experimented with, the system is still far from perfect. Among the problems are a lack of transparency of decision making processes for not directly involved citizens and a lack of access to validated state of the art knowledge for individual decision makers. One of the envisaged solutions is introducing software decision support into this international multi-stakeholder governance of nanomaterials.

This is of course not the first time such a tool is considered in supporting international norm creation. Cash et al. (2003) reviewed a large number of cases where science and technology have been mobilised for sustainability, linking knowledge to action. This is a different but overlapping problem statement from the central issue of international norm creation in the present paper. The main difference is that Habermas (explicitly) and Risse (implicitly) aim to contribute to solving the democratic deficit in the legislative process at international level, while Cash and others cited below take a more technocratic point of view and concentrate on how expertise can be fed into decision making.

The main issue Cash et al. perceive is organising boundary management between experts, policy makers and stakeholders, requiring a trade-off between salience (relevance to the policy making process), (scientific) credibility and legitimacy (to all stakeholders) of the knowledge. This calls for boundary management involving communication, translation and mediation in some form of institution that may utilise a “boundary object” such as co-development of a model or scenario by representatives of all three groups. The review reveals some unresolved issues:

- The demand side should be articulated better.
- The local, national and international level should be bridged.
- The existing models are valid for non-competitive sectors where existing knowledge should be translated into action.
- It is not clear how the private sector may be integrated in a public–private partnership (Cash et al. 2003).

These are key aspects in the case of international norm creation for nanomaterials. Despite several years of stakeholder and policy dialogue, it is not always clear which decision maker needs what kind of data. Governance initiatives are taken at national, transnational (EU) and global level, but not very well coordinated, according to some interviewed industrialists and regulators (Malsch et al. 2015). Companies manufacturing or handling nanomaterials operate in competitive markets which calls for confidentiality of some crucial information required by policy makers. Considerable data gaps exist and there is no consensus on what constitutes good data quality. The UK Voluntary Reporting Scheme for engineered nanoscale materials (2006–2008) demonstrates that engaging industry in a voluntary public–private partnership for norm creation on nanomaterials is complicated. In 2 years, only thirteen submissions were received.^11^ The mandatory French declaration of nanomaterials on the market introduced in 2012 was considerably more successful. They received 10,417 declarations in 2014 and 3409 in 2013, but this required prior adoption of a formal law.^12^

The Risk Governance framework developed and applied to nanotechnology by the International Risk Governance Council and the Dutch Health Council (Renn 2004; IRGC 2005, 2006; Health Council of the Netherlands 2006) distinguishes four categories of risk (simple, complex, uncertain and ambiguous), calling for increasing expert and stakeholder engagement. Natural nanostructured materials are classified as simple risks, and can be managed by agency staff and external experts. Engineered nanomaterials are classified as complex risks, for which the additional engagement of (unspecified) stakeholders is required. Active nanostructures and systems are classified as uncertain risks, calling for the additional engagement of industry and directly affected stakeholder groups. Large and molecular nanosystems are classified as ambiguous risks, calling for the additional engagement of the general public. The authors do not appear to have consulted stakeholders about their interest in engagement with each of the types of risk governance. Suggested methods for expert and stakeholder engagement do not include software tools or computer models.

Since 2006, the wiki-website Toxipedia^13^ attempts to bring together expert knowledge and stakeholder views on chemicals by inviting certified experts and interested lay persons to contribute different types of articles to an edited site. The information on nanotechnology is very limited and qualitative, restricting its usefulness for international norm creation. In the USA, the Environmental Protection Agency (EPA) has published several case studies applying Comprehensive Environmental Assessment to (sometimes remote) multidisciplinary expert and stakeholder engagement in decision making on the organisation’s nanomaterials research priorities: “Through structured decision-support methods stakeholders reach a collective judgment about priority areas of research to inform future risk assessment and management efforts for the nanomaterial of focus in a case study document”.^14^

The EU funded SUN project^15^ is currently developing a software decision support tool targeting nanomaterials, taking solicitation of stakeholder needs for decision support as starting point in the design process. This addresses Cash et al.’s first unresolved issue. We describe some of the features of this tool (currently under construction) and a thought experiment of a potential data-rich wiki-tool that are relevant for a decision support tool for better coordinated international governance of sustainable nanotechnology, addressing the other three issues.

### SUNDS tool design

Ongoing development of the SUNDS tool has given us an insight into some features of a tool that is suitable for international governance of nanotechnologies. SUNDS has been developed through a three-stage user elicitation process that involved potential users from industry, regulatory and insurance sectors from early stages of tool design (Malsch et al. 2014, 2015, the user workshop on 21 October 2014), which have played a significant role in defining the capabilities and features of the tool. The SUNDS is tailored to the European REACH regulation, which is considered to be the most comprehensive regulation balancing environmental risk and commerce of chemicals used in industry, and contains common elements with regulations of other countries such as various US regulations applying to engineered nanomaterials and nano-enabled products. SUNDS utilises multi criteria decision analysis (MCDA), which gives it two important capabilities for the governance of nanotechnology: (a) Technical criteria and stakeholder values can be integrated to support decision making about safety and sustainability of nanotechnology, (b) Uncertainty estimation techniques can be used to characterise the knowledge and data gaps associated with various criteria, and (c) Sensitivity analysis can identify the technical criteria and user values that affect the decision model the most (Subramanian et al. 2014, 2015). Thus, not only can scientific evidence and stakeholder values be integrated in norm creation, but users also have the choice to apply the precautionary principle to decisions concerned with the safe production, handling and disposal of nanomaterials. Further, uncertainty estimation and sensitivity analysis results can potentially contribute to reformulation of the framework and also serve as means for learning within the epistemic community.

SUNDS is based on a two-tiered framework with different complexities of tools and data requirements to cater to diverse users and different levels of data availability. Tier 1 is composed of the LICARA NanoSCAN, a deterministic tool developed specifically for small and medium enterprises (SME). The tool performs a semi-quantitative benefit-risk evaluation of nano-enabled products over their life-cycle and has low data requirements. Specifically, the model looks at environmental, social and economic benefits vs. ecological, occupational and consumer risks. Tier 2 of SUNDS includes the same components as Tier 1, but manifests a higher level of complexity and certainly has significantly higher data requirements. It comprises two modules: (a) risk control (RC) module, and (b) socioeconomic assessment (SEA) module. The RC module integrates quantitative ecological and human health risk assessment tools and facilitates users to select technological alternatives and risk management measures to reduce risks based on efficiency of mitigation, technological maturity and cost. The SEA module will compare the monetised benefits and costs of nano-enabled products with conventional alternatives. The SUNDS tool aims to evaluate the benefits and the risks of nano-enabled products along their life cycle. International governance of nanotechnology encompasses higher levels of social organisation and different governance concerns that can be addressed by further expanding the SUNDS tool.

### Option: a wiki-tool for global governance of nanotechnology?

As part of the stakeholder needs solicitation for the abovementioned tool, a roundtable was organised on “Computer guidance and decision making for safe and sustainable nanomaterials”.^16^ As a thought experiment, commentator Alfred Nordmann suggested introducing a wiki-like system supporting public collective decision making. In his view, this could address the problem that currently there is too much and too little information about risks of nanomaterials. So many factors are relevant. Human decision makers can’t take them all into account. The tool could function as a sort of extension or prosthesis of the mind. An individual decision maker can’t really think through all the relevant aspects and needs a machine to assist him or her. This is a form of e-science or simulation that is commonly used for processing information, but here also for supporting decision making. How would this tool work: if it is open access, people will be able to toggle the parameters and include their own weighing. They should be able to tamper with all the different elements of risk. The tool could help politicise science: use the tool to make a more democratic decision making on risk management (Nordmann, personal communication).

The interviews on stakeholder needs for the abovementioned tool design also addressed a similar potential role in international governance: “According to some interviewed policy makers, they are not likely to use software decision support tools in preparing political decisions on regulating nanomaterials, especially not in the short term. According to other stakeholders, policy makers could benefit from tools that indicate whether they should take action on adapting regulation, or that support international harmonisation of nanomaterials regulations” (Malsch et al. 2015).

This article explores the implications and feasibility of Nordmann’s suggestion for a potential role in democratising international governance of nanomaterials taking into account the views of industry, regulators, insurance company representatives and risk assessment specialists present in the user workshop on 21 October 2014.

### Supporting access to the governance arena for transnational actor networks

Following Risse (2002), three overlapping types of transnational actors are already active in the international nanomaterials governance arena in addition to regulators from states and international organisations: epistemic communities, multinational companies and advocacy networks. As a thought experiment, how could each of these actors make use of a wiki-tool and what role could such a tool have in democratising the norm creation process?

#### Epistemic communities

The major dynamics of epistemic policy coordination are uncertainty, interpretation and institutionalisation (Haas 1992, p. 3). In the case of nanomaterials, a professional epistemic community has formed consisting of researchers from traditional risk assessment disciplines (c.f. Klaessig 2014) whose common policy enterprise is reducing uncertainty about risks of engineered nanomaterials in order to facilitate adaptation or creation of international chemical norms. He also reveals the existence of differences in interpretation of scientific results and even of what constitutes good science, and institutionalisation of an “official science” epistemic community with its common policy enterprise aiming to regulate nanomaterials at the expense of normal science in Europe and the OECD.

The current uncertainty in governance of nanomaterials is even broader than Klaessig envisages, extending to the selection of relevant scientific disciplines. Most often, this includes expertise on “Exposure through the Life Cycle, with Material Characterization, Ecotoxicity Testing and Predictive Models, with Material Characterization, Predictive Modelling for Human Health, with Material Characterization, Databases and Ontologies, Risk Assessment and Risk Management and Control”^17^ or similar environment, health and safety and life cycle assessment topics. In some cases more areas of research are taken into account including economic, ethical, legal and social aspects, but the uncertainty of what constitutes salient, credible and legitimate data from those areas is even bigger than for the more traditional disciplines. In addition, many published results are qualitative and therefore hard to integrate in a computer model.

Notwithstanding these practical issues, the proposed wiki-tool should collect data resulting from studies in a variety of scientific disciplines including environment, economic and social aspects. The people who are expected to insert facts are therefore experts from these different disciplines who can be considered members of this emerging epistemic community. The design of the tool could influence which experts are considered members of the epistemic community of sustainable nanomaterials. The most restrictive option would be to ask the prospective user for professional credentials before deciding which kind of input he or she can insert: data and values or only values.^18^ This presupposes that the manager of the tool has decided beforehand which areas of research are relevant and which are not. A suggestion made by the stakeholders whose views have been solicited is to introduce quality control on the data before allowing any user to insert new data resulting from scientific studies. Thereby “official science” is distinguished from other normal science that also has been subject to peer review and hence is expected to correspond to commonly agreed scientific quality standards. The most open option would be to allow anyone to insert and correct data he or she considers relevant, relying on the self-correcting ability of the emerging epistemic community without predetermining what is to be considered valid data and who is considered to be a member.

The discussions during the stakeholder workshop reveal a different structure of the emerging epistemic community interested in international risk governance of nanomaterials: the participation of industrial researchers in addition to academics from universities and public research organisations. The contribution of industrial researchers is essential for data on emissions and production volumes not available to academics. Intermediaries such as companies marketing nanomaterials can contribute such data, provided solutions are found to protecting proprietary data and sharing the costs of testing.

Intermediaries, associations and consortia or networks are commonly used to generate data for norm creation in a cooperative effort. Incentives may be:

- That profitable membership requires submission of data
- Support offered by the intermediary (e.g. exchange) or association in data collection, cooperation in subsidised research consortia
- Reduction in insurance premiums—insurers would be interested in a system that reflect the emergence of risks that would call for increases in insurance premiums
- Public image of the (large) company (sustainability)
- Communication with clients and partners in value chain (SMEs)
- Improving its own product
- Training by researchers and consultants how to use the tools

Barriers to participation of MNCs in the epistemic community include:

- Industrial users may require protection of proprietary data costing a lot of money
- Industry wants to avoid giving away expensive data to competitors—need accommodation for sharing this
- Nobody wants to take the lead in opening up the information
- Design and data requirements of SMEs and researchers clash
- Industrial users may lack the skills to use the tools

MNC overlap with epistemic communities, because they employ risk assessment specialist carrying out tests of nanomaterials in the company’s R&D and manufacturing. They are encouraged by policy makers to share these test results with the international community to facilitate the legislative process. A bottleneck hampering such contributions is the need for protection of proprietary data and to ensure that the costs are shared by all competitors and no one is given a free ride. Is it possible to address this issue specific to public–private partnership during the norm creation phase by smart design of the wiki-tool or is a formal legislative framework a prerequisite?

#### (Multinational) companies

A variety of companies participates or contributes to the value chain from nanomaterials R&D and production all the way to the waste processing stage of products incorporating nanomaterials. This includes large (multinational) companies as well as SMEs, service providers such as instrument manufacturers, banks and insurance companies and industrial associations. As discussed above some of those companies actively engage in dialogue on international norm creation, either directly (mostly associations and large companies) or indirectly through participating in working groups organised by associations (see also Malsch et al. 2015).

On the other hand, the companies are also stakeholders with interests they want to be taken into account by legislators. The discussions in the workshop reveal distinct subgroups with different interests, priorities and requirements for the tool. In particular, SMEs require tools that are user friendly and require little investment of time and researchers. Lack of knowledge on nanosafety inhibits innovation in large industry applying nanomaterials. Companies marketing nanomaterials have an interest in establishing their commercial value. Inside companies, workers and employers have overlapping but different interests in risk management of nanomaterials. Workers have a direct interest in protecting their own safety.

The envisaged wiki-tool would be a MCDA based Decision Support Tool that not only models the causal relationships between input data and output criteria that should be taken into account in decision making, but also foresees the introduction of weights reflecting different stakeholder groups preferences on more or less important criteria. These weights could be introduced in several ways. One way is by elicitation of average weights of the different user groups of the tools (e.g. industry as foreseen primary users), and fixing these weights in the system. Another way would be to include these average weights as default values in the tool, but allowing users to insert their own weights and compare those with the average of their own and other stakeholder groups. The second option would enhance transparency of how different values and preferences may influence international norm creation on nanomaterials.

#### Insurance companies

As argued in the section on nanotech sustainability from insurability, insurance companies play the role of proxy regulators during the international norm creation phase. The discussions during the workshop suggest that they need data to support insurance decisions (including estimates of uncertainty) such as occupational health and safety and catastrophic events (e.g. large scale occupational disease that manifests itself after 20 years or so). They currently seldom make use of decision support tools or computer models in supporting their decision making on insurance policies. One insurance company represented in the user workshop has used PraediCAT,^19^ an analysis of nanomaterials citations in 250 medical journals, and is continuing discussions on how this tool can be used effectively. This suggests that there may be interest in similar tools. In the proposed wiki-tool, insurance companies could be interested to insert their weights and compare them with the averages of their own and other stakeholders in order to support their insurance strategy.

#### Regulators

On the regulatory side, national, transnational (e.g. EU) and international policy makers (e.g. OECD) participate in international norm creation, including representatives of states as well as the European Parliament. Authorities participate in norm implementation. During norm creation, regulators are interested in a category approach enabling read across to drive risk assessment outcomes for risk prioritisation and identification of testing needs. They would be interested in a system that reflect the emergence of risks that would call for new regulation. (based on discussions in the workshop and Malsch et al. 2015).

The preferences or weights of different criteria of these regulators as well as of authorities overseeing norm implementation will be inserted in the system or may be inserted by regulatory users as in the case of company representatives.

#### Advocacy networks

Relevant advocacy networks tend to focus on a single legislative phase. In dialogue on international governance of nanomaterials and nanotechnologies, trade unions and their European and international associations, environmental groups and networks, consumers and patients associations, animal rights activists and other interest groups have been participating since around 2003. In many cases these groups have cooperated with academics including social scientists, philosophers and risk assessment specialists because of lacking technical expertise in the organisation. A prime example is the EU funded project NanoCAP (2006–2009), where research groups trained civil society organisations enabling them to formulate positions and participate in the dialogue on governance of nanomaterials and nanotechnologies.^20^

The stakeholder needs elicitation in this SUN project has not covered advocacy networks. An interviewed regulator is interested in a tool “that can make it easier for NGOs and watchdogs to monitor compliance of companies to the norms during (the future phase of) implementation.”

Other studies may suggest how a wiki-type software decision support tool could enable advocacy networks to contribute to the current norm creation and whether there would be any interest in such contributions from their side. For example, Invernizzi (2012) cites a trade union representative who complained that they had difficulties negotiating about nanotechnology regulation because the discussion was limited to technical aspects, and that they had difficulties hiring technical experts. Malsch (2013) reports on interviews with several stakeholders including civil society representatives about their need for education and training in nanotechnology. Most of them were only working on nanotechnology issue part of their time for a limited period, and were interested in learning about “nanoscience, nanomaterials, nanorisk assessment, and legal and social aspects of nanotechnology” in general and in “specific aspects of nanotechnology that enter their agenda for a short time, and then shift focus to other topics that may not be related to nanotechnology. Examples include graphene, nanoparticles in waste, etc.” Preferred teaching methods include: “specific courses, conferences, or projects for the staff, training on the job/learning by doing, and networking/asking experts inside/outside the organisation” (Malsch 2013). Computer models or software decision support tools were not part of that study nor were they mentioned spontaneously.

#### Supporting global citizenship

Returning to Habermas’ vision on a dual national and global citizenship for all, calls for discussion of another type of actor that has so far been excluded from the analysis in this article: the elected representatives of citizens. Some initiatives and dialogue projects on nanotechnology have attempted to foster direct democracy (projects have been reviewed in Malsch et al. 2012). However, Habermas presupposes the model of a representative national democracy and expands this to the trans- and international level. Could the proposed wiki-tool support decision making on new or adapted international norms at the level of the UN General Assembly or any future Global Parliament? The history of national and European Parliamentary Technology Assessment organisations demonstrates the complexity of raising the awareness of politicians to issues related to emerging technologies and of offering them salient, credible and legitimate information in time for adoption of legislation.^21^ Whether politicians would be interested in a software decision support tool has not been investigated, but interviewed policy making preparing political decisions do not consider such tools appropriate (Malsch et al. 2015).

In a direct democracy scenario on the other hand, the envisaged wiki-tool could offer any interested individual with internet access the opportunity to understand the criteria and values taken into account in international norm creation on nanomaterials, but also to influence the values underlying this process by inserting his or her own values in the weights of the tool. Though there would still be distinct roles for experts contributing scientifically validated data and lay persons, the latter would be able to influence the values attributed to different criteria taken into account in norm creation.

## Grounded theory for international governance of nanomaterials

**Fig. 4:**
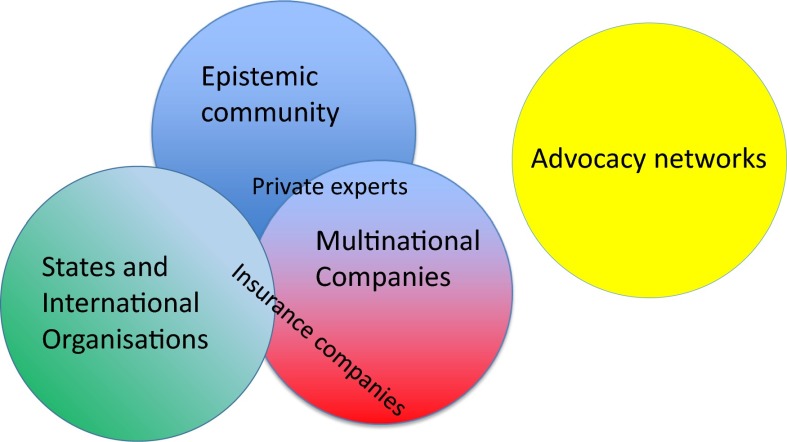
Interrelationships of transnational actors, states and international organisations in international governance of nanomaterials

Risse’s discussion of the role of transnational actors in international norm creation inspires a search heuristic to discover the potential roles of epistemic communities, multinational companies, advocacy networks and states and international organisations in international governance of nanomaterials potentially aided through decision support tools. Constant comparison with discussions during a stakeholder workshop and literature reveal that these communities are internally structured and consist of different kinds of actors with distinct roles and interests, and that the main categories overlap, with dual roles for individual researchers and professionals as well as organisations. Figure 4 aims to capture this internal structure of the transnational actor networks and their relationships with states and international (governmental) organisations in international governance of nanomaterials. Overlaps between epistemic communities and multinational companies take the form of industrial experts contributing to the generation and collection of data for risk management and sustainable manufacturing of nanomaterials, either directly as employees of their company or mediated through companies specialising in marketing of nanomaterials, industrial associations or other networks. Overlaps between multinational companies and states and international organisations take the form of insurance companies acting as proxy regulators. Overlaps between epistemic communities and states and international organisations take the form of researchers in public risk assessment research centres with a role in the regulatory process. The discussions during the stakeholder workshop revealed an underlying conflict between governmental policy makers and academic researchers that corroborates the findings of Klaessig (2014). The selection of good quality data can either be based on protocols prescribed by the EU and OECD or on expert judgement. The design of a decision support tool can offer an arena for resolving this conflict by incorporating the protocols as well as the option to insert changes based on progress in the scientific state of the art.

The discussions during the workshop did not involve representatives of advocacy networks, therefore these are set apart in this representation of international governance of nanomaterials. Further research is needed to reveal possible relations between advocacy networks and other transnational actors.

Two stages of the international norm cycle proposed by Risse (2002) play a role in the discussions during the workshop: governance during norm creation and compliance during norm implementation. These stages appear to be running in parallel: while the dialogue on creation of new norms and the applicability of existing norms is still ongoing, established or recently adapted norms are already governing nanomaterials.

During the norm creation phase, regulators are under pressure to regulate nanomaterials in the absence of the required relevant data. The precautionary principle or a precautionary approach govern the handling of nanomaterials. Governance during this phase includes three aspects: a legal review of the current regulatory framework, a ranking of policy options and a series of interpretative norms that can be used as quasi-directive as it can be used for risk management that can be transposed to nanomaterials. Regulators are interested in support for decisions on risk management of nanomaterials. MCDA may offer opportunities according to some interdisciplinary regulators, but is contested by others requiring hard data as basis for decision making.

The fledgling nanomaterials producing industry is called upon to engage in risk assessment supporting the norm creation process, but this is not a traditional role for industry. In addition, industry in general is expected to use the tool in (non-mandatory) sustainable manufacturing to select less hazardous materials early in R&D. Some large companies consider this in their own self-interest, while most are not intrinsically motivated for this. Intermediaries and insurance companies are already collecting data from their members that could be inserted in the tool and contribute to supporting the creation of international evidence based norms. They may offer support and benefits to the companies. Industry could also be pressed into using non-regulatory risk assessment through threats of litigation on manufacturing as developed in the USA. Suppliers and partners in the value chain as well as industrial associations and consortia can offer support or a platform for generating common data sets.

Most SMEs (and large industry) will not use a tool unless it is compulsory, limiting its use to the norm implementation phase. Regulatory compliance is very important for them. The tool could be used to demonstrate compliance with existing legislation including REACH, cosmetics and biocides regulations in Europe.

The discussions during the workshop suggest that in the long term, a decision support system may contribute to epistemic coordination through uncertainty reduction (Haas 1992) by gradually replacing defaults plus an uncertainty estimate with hard data. The developers of the tool can incorporate data about selected nanomaterials, but later users may insert data on a broader range of (nano) materials. The design of the wiki-tool must be open to such user input. Types of data include: Risk and Environmental Impact Assessment module (which in turn includes sub-modules on ecological risk assessment, human health risk assessment and environmental impact assessment), Economic Assessment Module and Benefits Assessment module. Data on hazards as well as exposure and dose–effect models can be incorporated by the tool designers. The tool design may incorporate absolute or comparative assessment of technological alternatives depending on data availability and user requirements. Data on nanomaterial, risk management and production volumes in the value chain could also be incorporated later on by companies involved in marketing nanomaterials. The costs of insurance could be incorporated as an indicator of economic aspects by later users of the tool.

Mixing values with data is problematic because of legal and political reasons. It is important to clearly separate them in the tool. Examples of values include the following: The definition of nanomaterial imposed by regulators determines the scope of what must be tested. This is a political more than a science-based decision. Data quality can be determined through protocols imposed by regulators and scientific expert judgement (combining values from different stakeholder groups). The precautionary principle or approach is a core but controversial concept in governance of nanomaterials, that should not be incorporated in the tool design. Instead, uncertainties should be made explicit allowing users to be as precautionary as they wish. The level of acceptable risk for the different endpoints can either be based on current regulator preferences or on scientific expert community judgement. The tool designers can insert a fixed set of endpoints based on regulation and let the scientific community define additional endpoints. Which weights will be included for the different endpoints as default and which options the user will have is an open question.

## Conclusions

Recall the first central research question: “What can theories of international relations and deliberative democracy contribute to understanding multistakeholder governance of emerging technologies?”

Risse (2002), Haas (1992) and others offer the conceptual tools to understand the roles that are currently played by epistemic communities (including academic and private sector researchers from a variety of disciplines), (multinational) companies playing different roles in the value chain for nanomaterials and advocacy networks targeting different phases in the legislative framework in addition to states and international organisations in this governance process at international level. The workshop discussions reveal an internal structure of each of these categories and overlap between them. Habermas, Rawls and others offer the visionary institutional framework of how anyone in a dual or even triple role of national, (transnational) and global citizen could contribute to international norm creation.

In response to the second central research question: “What role could software decision support play in democratising such governance?”, there appears to be room for wiki-like software decision support tools in the international governance of nanomaterials. This three-stage process consists of past agenda setting, current norm creation and current as well as future norm implementation. A well-designed wiki-tool could bring together test data and values from all experts and stakeholders and increase the transparency of the scientific data and values taken into account in international norm creation. Now and in the future, the tool could be used by all to demonstrate, verify and monitor compliance with the norms.

Comparing the sketched international governance context and the wiki-option with the current design of the SUNDS tool highlights the following discussion points for further consideration:

How can citizens be empowered in international governance of nanomaterials (input legitimacy, output legitimacy and governance during international norm creation) through decision support?

What would be the optimal democratic and technically feasible option to ensure good data quality and the protection of proprietary data while allowing anyone to toggle with the weights?

Is it possible to allow users to insert their own weights and compare them with the average of their own peer group and other stakeholders?

Could the design be flexible enough to accommodate different emerging legislative frameworks (including a new “Lex Specialis” as well as distinct legal frameworks for each of the four phases in the legislative cycle and for each type of consumer product)?

## Footnotes

1 http://link.springer.com/journal/11569.

2 The relevance of Rawls’ Theory of Justice for responsible governance of nanotechnology at global level is discussed in Malsch (2011).

3 The participation model of procedural justice holds that procedural fairness requires that those affected by a decision have the option to participate in the process by which the decision is made. There is a deep, constitutive connection between participatory process, correct outcomes and distributive justice.

4 1. A belief in deliberation with decisions reflecting public regarding reasons. 2. A true commitment to citizenship and wide spread participation by the public. 3. A commitment to the idea of *agreement* as a tool for regulation/governance—that is, agreement among equal citizens through deliberation concerning public regarding reasons as opposed to conclusions which note the different perspectives of disagreeable people. 4. Political equality which prohibits disparities in influence by different social groups.

5 1. The maximisation of liberty ( here liberty refers to the right to participate in deliberation) subject only to such constraints as are essential for the protection of liberty itself; 2. Equality of the basic liberties of social life and the distribution of social goods, subject only to the exception that inequalities may be permitted if they if they produce the greatest possible benefit for those least well off in a given scheme of inequality - “the difference principle” ( for our purposes equality places all stakeholders on an equal footing via their representative); 3. Fair equality of opportunity and the elimination of all inequalities of opportunity based on birth or wealth.

6 In “the constitutionalisation of the international law and the problems of legitimation of the constitutional global society”).

7 There are also bilateral dialogue platforms including most notably the EU-US Communities of Research in Nano-EHS: http://us-eu.org/. Such bilateral platforms also play a role in negotiating an international consensus, but do not have the authority to impose international norms, as is the case with the OECD Council of Ambassadors for OECD Member States.

8 IOMC participants are Food and Agriculture Organization of the United Nations (FAO), International Labour Organization (ILO), United Nations Development Programme (UNDP), United Nations Environment Programme (UNEP), United Nations Industrial Development Organization (UNIDO), United Nations Institute for Training and Research (UNITAR), World Health Organization (WHO), World Bank and Organisation for Economic Cooperation and Development (OECD). Info: http://www.who.int/iomc/en/.

9 The SAICM is a policy framework adopted by the International Conference of Chemicals Management (ICCM) in 2006 for ensuring that, by 2020, “chemicals are produced and used in ways that minimse significant adverse impacts on the environment and human health” (Johannesburg World Summit on Sustainable Development, 2002). SAICM’s secretariat is hosted by the UN Environmental Programme UNEP. Info: http://www.saicm.org/index.php?option=com_content&view=article&id=456&Itemid=689.

10 http://www2.unitar.org/cwm/publications/Nano.aspx.

11 http://archive.defra.gov.uk/environment/quality/nanotech/policy.htm (Accessed 06-02-2015).

12 https://www.r-nano.fr/.

13 http://toxipedia.org/display/toxipedia/Toxipedia.

14 http://cfpub.epa.gov/ncea/CFM/nceaQFind.cfm?keyword=Nanomaterials.

15 www.sun-fp7.eu.

16 During the S.NET 6th Annual Meeting “Better Technologies with No Regrets?”—21–24 September 2014, Karlsruhe Germany, www.itas.kit.edu/snet2014.

17 The current US-EU Communities of Research Groups contributing to the EU-US collaboration on nanosafety research, http://www.sun-fp7.eu/events/upcoming-events/eu-us-bridging-nanoehs-research-efforts-a-joint-workshop-2015/ (Accessed 12-02-2015).

18 The Toxipedia platform uses such a selection procedure.

19 www.praedicat.com.

20 www.nanocap.eu (accessed 12-02-2015).

21 The first was the Office of Technology Assessment in the United States, which has been closed years ago. Currently the US Government Accountability Office GAO is an associate member of the European Parliamentary Technology Assessment (EPTA) network. At the European Parliament and several national parliaments such offices are still operational. C.f. http://www.eptanetwork.org/.
